# Concurrent IgA Nephropathy and Membranous Nephropathy, Is It an Overlap Syndrome?

**DOI:** 10.3389/fimmu.2022.846323

**Published:** 2022-03-11

**Authors:** Jia-Wei He, Dong-Feng Cui, Xu-Jie Zhou, Pei Chen, Yang Li, Xue Zhang, Yan-Na Wang, Ting Gan, Li-Jun Liu, Su-Fang Shi, Li Zhu, Ping Hou, Ji-Cheng Lv, Hong Zhang

**Affiliations:** ^1^ Renal Division, Peking University First Hospital, Peking University Institute of Nephrology, Key Laboratory of Renal Disease, Ministry of Health of China, Key Laboratory of Chronic Kidney Disease Prevention and Treatment (Peking University), Ministry of Education, Beijing, China; ^2^ Renal Division, The Third People’s Hospital of Zhengzhou, Zhengzhou, China

**Keywords:** IgA nephropathy, primary membranous nephropathy, galactose-deficient IgA1, anti-phospholipase A2 receptor, polygenic risk score

## Abstract

IgA nephropathy (IgAN) and membranous nephropathy (MN) are common glomerulonephritis, the presence of which in the same patient– concurrent of IgAN and MN (cIgAN/MN) has been described occasionally. This study aims to show clinical-pathological features of cIgAN/MN and attempts to suggest underlying pathogenesis using disease-specific biomarkers and a genomics approach. This retrospective cohort study described the clinical and pathological data from 137 patients with cIgAN/MN diagnosed in Peking University First Hospital from 2005 to 2019. One hundred primary IgAN and 100 MN cases were randomly selected as disease controls between the same time interval. Moreover, disease-specific biomarkers and polygenic risk score models were conducted to reveal the underlying pathogenesis. The median age of the cIgAN/MN cases was 45-year-old, and 46% were women. Compared to IgAN, patients with cIgAN/MN had a higher level of 24-hour proteinuria excretion but lower microscopic hematuria. They had a lower median level of galactose-deficient IgA1 (Gd-IgA1, 4.00 versus 5.45 μg/ml, *P*=0.002) as well as the standardized genetic risk scores of developing IgAN (GRSs: 0.05 versus 0.68, *P*<0.001). Compared to MN, patients with cIgAN/MN had a lower proportion of nephrotic syndrome and a lower level of albumin-to-creatinine ratio. However, the 24-hour proteinuria levels, serum lipid profiles, proportion of hypertension, and pathology classification were similar. Patients with cIgAN/MN had lower levels of plasma autoantibodies against the M-type transmembrane phospholipase A2 receptor (PLA2R) (11.23 versus 36.59 U/ml, *P*=0.005). Intriguingly, there were no statistical differences in standardized GRSs of developing MN between them (2.77 versus 3.02, *P*=0.326). Compared to IgAN, cIgAN/MN may lean towards MN more according to clinical-pathological features, disease-specific biomarker levels, and disease-specific genetic risk scores.

## 1 Introduction

Immunoglobulin A nephropathy (IgAN) and membranous nephropathy (MN) are two types of common glomerulonephritis (GN) worldwide. It was reported that IgAN is the most common GN in patients less than 59-year-old, and MN is the most frequently observed GN in patients at age ≥60 years (1). These two diseases differ in clinical features, pathology, treatment, and prognosis. IgAN is more unlikely to develop hypo-albuminemia but more likely to present episodes of gross hematuria. IgAN can only be diagnosed with a kidney biopsy (2). However, a kidney biopsy may not be required to confirm the diagnosis of MN in patients with a compatible clinical and serological presentation like antibodies against PLA2R according to the 2021 KDIGO guideline (3, 4). Proteinuria reduction to <1g/d is a surrogate marker of improved kidney outcome in IgAN, and immunosuppressive drugs should only be considered in patients with IgAN who remain proteinuria >1g/24h despite at least 90 days of optimized supportive care. Differently, immunosuppressive therapy may not be required in patients with MN when proteinuria <3.5 g/d and eGFR >60 ml/min/1.73 m^2^. Thus, elucidating the underlying disease is pivotal in guiding management and treatment decisions, which seems to be difficult when two diseases coexist in the same patient.

It is suggested that concurrent IgAN and MN (cIgAN/MN) in the same patient is rare, but more and more cases have been reported since 1983 (5–11). From the literature, all the patients had hematuria and nephrotic range proteinuria. In our previous reports on clinical-pathological features of 26 patients with cIgAN/MN, we observed that these patients displayed similar clinical features with MN patients and milder pathological lesions than IgAN patients (12), and they had comparable serum levels of Gd-IgA1 with IgAN, but lower detectable serum levels of anti-PLA2R compared with MN. These patients showed characteristics of both diseases. We thus suggested that cIgAN/MN might result from superimposed MN on a background of preexisting mild IgAN. However, the limitations of the previous studies are the small sample size and lack of pathogenesis investigation.

Thus, the aim of this study is to show clinical-pathological features with a larger sample size in 137 patients of cIgAN/MN and attempt to suggest underlying disease pathogenesis using disease-specific biomarkers and a genomics approach. Any attempts to address the issue should be of clinical relevance as different treatment strategies may lead to different prognoses.

## 2 Article types

Original Research.

## 3 Materials and Methods

All the study protocols complied with the principles of the Declaration of Helsinki and were approved by the Ethics Committee of Peking University First Hospital (Institutional Review Board number: 2021[Y148]). Written informed consent was obtained from all the participants.

### 3.1 The Process to Select Patients With cIgAN/MN

A retrospective cohort study (2005-2019) was established in Peking University First Hospital to study the features of patients with concurrent cIgAN/MN. First, patients diagnosed with IgAN from November 2005 to June 2019 were included in the study cohort (n=10387). The diagnosis of IgAN was based on dominant staining for IgA in the glomerular mesangium on immunofluorescence microscopy and electron-dense deposits in the mesangium on electron microscopy. Patients who were secondary IgAN, such as Henoch-Schonlein purpura (IgA vasculitis), systemic lupus erythematosus, thin basement membrane disease, Alport syndrome, Fabry disease, or without biopsy samples in electron microscope were excluded from our study (n=912).

**Figure 1:**
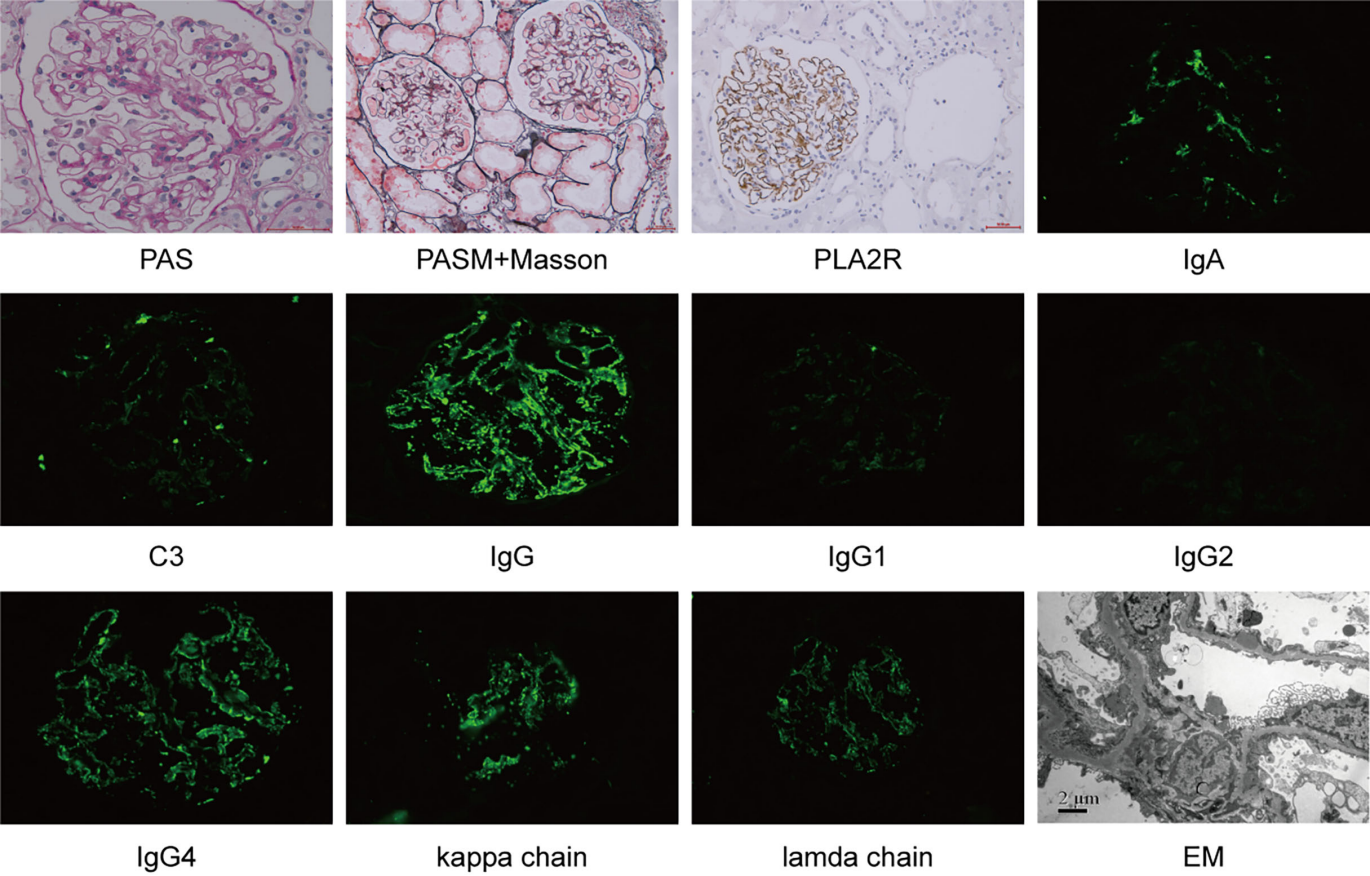
Kidney biopsy from a case with concurrent IgAN and MN. PAS, Periodic Acid-Schiff; PASM, periodic acid-silver methenamine; EM, electron microscope.

**Figure 2:**
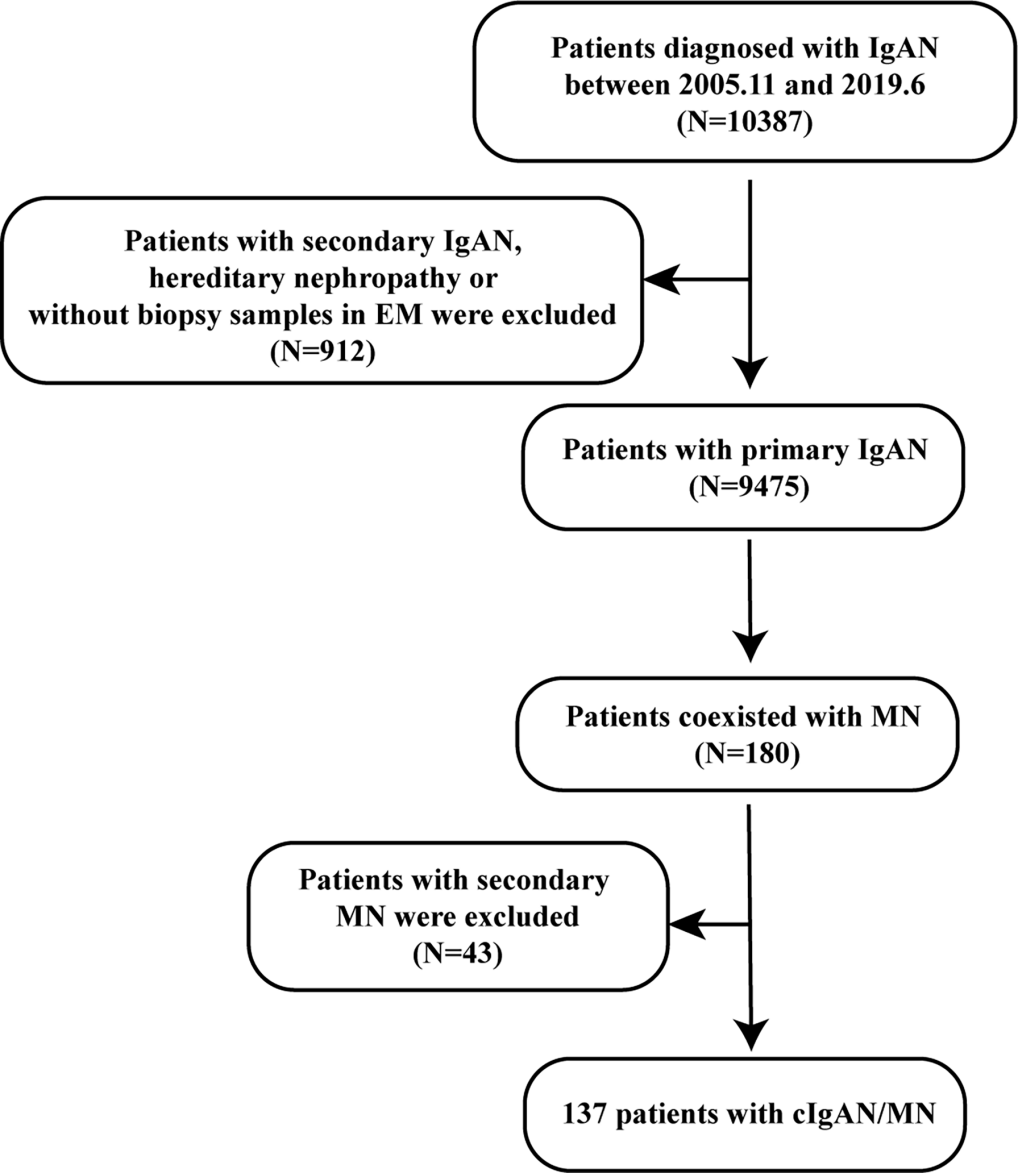
Flowchart of the recruitment process.

Secondly, we screened those patients with concurrent MN (n=180). The diagnosis of cIgAN/MN was additionally confirmed by kidney biopsy with membranous thickening of the glomerular capillary wall; dominant staining for IgG, C3 in glomerular capillary walls on immunofluorescence microscopy; the existence of subepithelial electron-dense on electron microscopy (**Figure 1**). Patients who were secondary MN, like autoimmune disease (lupus erythematosus), infection with hepatitis B, hepatitis C, or syphilis, certain medications (gold/mercury salts and nonsteroidal anti-inflammatory drugs) or solid cancerous tumors or blood cancers were excluded from our study (n=43). The flowchart of the recruitment process was displayed in **Figure 2**.

### 3.2 The Selection of the Disease-Control Groups

Meanwhile, we searched our database for disease-control groups from the same time period. One hundred patients with primary MN and 100 with primary IgAN were retrieved using the stratified random sampling method as disease controls based on the year of hospital admission.

### 3.3 General and Clinical Information of All the Enrolled Patients

Demographics and clinical features at the time of renal biopsy performance included age, gender, blood pressure, urinary sediment microscopy, 24-hour urine protein excretion, serum levels of IgA and IgG, uric acid, lipid profiles, and creatinine. Hyperlipidemia was defined as the total cholesterol above 6.2 mmol/L or triglycerides above 2.3 mmol/L. The estimated glomerular filtration rate (eGFR) was calculated by the Chronic Kidney Disease Epidemiology Collaboration equation (13).

### 3.4 Pathological Features

All the kidney sections were processed for immunofluorescence examination, light microscopy, and electron microscopy. Pathological data were also recorded for each patient, including the degree of mesangial cell proliferation, the proportion of glomeruli with cellular/fibro cellular/fibrous crescents, the intensity of immunofluorescence staining (IgG/C3/IgA), and electron-dense deposits on the mesangial area or glomerular basement membrane (GBM). Because in the original development of Oxford classification, those with secondary causes of mesangial IgA deposits or those with comorbid conditions were excluded. The Oxford MEST scores may be not proper in evaluating pathology lesions in patients with cIgAN/MN, which were not reported here. The pathological stages of MN were based on the electron microscopic findings, referring to the immune deposits in the GBM, GBM reaction to the deposits, and resolution of glomerular injury with resorption of the deposits (14).

### 3.5 Assay of Gd-IgA1 and IgA1

Levels of plasma Gd-IgA1 (1:200 dilution) were detected using the KM55 ELISA kit (27600, Immuno-Biological Laboratories, USA). All the experiments were done according to the manufacturer’s instructions. Moreover, plasma IgA1 levels were determined using capture ELISA. High-absorption polystyrene plates (Thermo, USA) were coated with 2.5 mg/ml F(ab’)2 fragment of goat anti-human IgA (Jackson ImmunoResearch, USA) overnight at 4°C. After washing and blocking with 1% bovine serum albumin in PBS with 0.1% Tween, the diluted plasma (1:80000) was added for incubation. The diluted plasma was then detected by the anti-human IgA1 horseradish peroxidase-conjugated antibody (25-783-72807, Gentaur, Belgium). The optical density at 450 nm was measured after the tetramethylbenzidine liquid substrate system was applied.

### 3.6 Assay of Anti-PLA2R Antibodies

Circulating anti-PLA2R antibodies were detected by using commercial ELISA kits (EUROIMMUN AG, Lübeck, Germany). The results were negative for <20 units (U)/mL and positive for ≥20 U/mL (15).

### 3.7 Single Nucleotide Polymorphisms (SNPs) Selection and Genotyping

Disease-specific genetic risk scores were calculated to deepen our understanding of the pathogenesis of cIgAN/MN. As previously reported, the IgAN risk score equation was based on the 15 SNPs associated with IgA nephropathy in the analysis of 20,612 individuals from 14 international case-control cohorts of European and Asian ancestry (16). The risk score was standardized using genotypes of 1,050 individuals from 52 worldwide populations included in the Human Genome Diversity Project (17). The MN risk score equation was derived from GWAS analysis of 12,820 individuals (3,782 primary MN and 9,038 controls), including 4,841 individuals of East Asian ancestry (1,632 cases and 3,209 controls) and 7,979 individuals of European ancestry (2,150 cases and 5,829 controls). We adopted the East Asians risk score, which is calculated separately for East Asians and standard-normalized using genotypes of healthy ancestry-matched controls (18).

Peripheral blood samples were collected from participants using anticoagulant EDTA. We obtained the genomic DNA from peripheral blood leukocytes using the salting-out method. Genotyping was performed using the Kompetitive Allele Specific PCR (KASP) assays (Compass Biotech, Tianjin, China) among 132 patients with cIgAN/MN, 96 with MN, and 98 with IgAN.

### 3.8 Genetic Risk Score Assessment

Individuals with 100% non-missing genotypes across all the scored loci were analyzed. The weighted genetic risk score (GRS) assessment was adopted to evaluate individual disease risk. The weighted-GRS utilized the allelic odds ratios to account for the strength of the genetic association within each allele because alleles might have different odds ratios. The weighted GRS was the weighted sum of risk allele counts, where the weight for each SNP was the natural log of the odds ratio. Details about the calculation formula were derived from http://www.columbiamedicine.org/divisions/kiryluk/resources.php.

### 3.9 Follow-Up Data

Follow-up was defined as the interval between renal biopsy and the last outpatient visit. Full details about the follow-up data, such as general clinical course, medication history, renal function, and urinalyses, were recorded. Time-averaged proteinuria (TA-proteinuria) was calculated as the weighted mean of all the 24-hour urine protein excretion measurements during follow-up, with the weight representing the time elapsed since the previous measurement (19). Patients were also classified according to the magnitude of TA-proteinuria (>1.0 or ≤1.0 g/d). Complete remission was defined as urinary protein excretion <0.5 g/24 h confirmed by two values at least one month apart, accompanied by a normal serum albumin concentration and normal serum creatinine level. Partial remission was defined as urinary protein excretion <3.5 g/24 h and reduced by at least 50% from peak values, accompanied by an improvement or normalization of the serum albumin concentration as well as stable serum creatinine.

### 3.10 Statistical Analysis

Continuous variables in this study were compared using an unpaired *t*-test or analysis of variance (ANOVA) between groups if the variables were normally distributed; otherwise, a Mann–Whitney U test or Kruskal-Wallis test was performed. Categorical variables were compared using the chi-square test or Fisher’s exact test. Cumulative proteinuria remission rates were calculated according to the Kaplan-Meier method (log-rank test). The statistical analysis was performed with the STATA 15.0 (Texas, USA). A two-tailed *P*-value <0.05 was considered statistically significant.

## 4 Results

### 4.1 Demographics

From November 2005 to June 2019 in Peking University First Hospital, a total of 9475 primary IgAN patients and 9335 MN patients were diagnosed, of which 137 patients met the diagnostic criteria of cIgAN/MN, accounting for 1.45% and 1.47% of the Chinese patients with IgAN and MN, individually.

Patient demographics were summarized in **Table 1**. Patients with cIgAN/MN had a median age of 45 years. Patients with cIgAN/MN were older than IgAN (35 years, *P*<0.001) but younger than MN (50.5 years, *P*<0.001), who were randomly selected at the same time interval. Among 137 cases with cIgAN/MN, there were 74 males (54.01%) and 63 females (45.99%). No gender distribution difference was observed among IgAN, cIgAN/MN, and MN.

**Table 1:** Demographics and clinical features of patients with MN, cIgAN/MN, and IgAN.

Characteristics	MN (n = 100)	cIgAN/MN (n = 137)	IgAN (n = 100)	*P* (cIgAN/MN vs. MN)	*P* (cIgAN/MN vs. IgAN)
Demographics					
age (y)	50.50 (41.00, 62.50)	45.00 (36.00, 55.00)	35.00 (29.00, 43.00)	<0.001	<0.001
female n(%)	42 (42.00%)	63 (45.99%)	47 (47.00%)	0.542	0.877
clinical features					
nephrotic syndrome n(%)	72 (72.00%)	65 (47.45%)	10 (10.00%)	<0.001	<0.001
proteinuria (g/24h)	4.63 (3.00, 7.89)	3.96 (2.06, 6.00)	1.22 (0.65, 2.75)	0.060	<0.001
albumin-to-creatinine ratio (mg/g)	1552.31 (391.77, 3070.74)	617.64 (254.30, 1764.42)	458.04 (237.33, 807.56)	0.032	0.247
serum albumin (g/L)	25.35 (21.80, 30.15)	28.10 (22.50, 33.80)	38.40 (35.05, 41.75)	0.025	<0.001
hyperlipidemia n(%)	81 (82.65%)	92 (78.63%)	30 (30.30%)	0.459	<0.001
total cholesterol (mmol/L)	6.85 (5.78, 8.50)	7.20 (5.70, 8.62)	4.61 (4.05, 5.25)	0.977	<0.001
triglycerides (mmol/L)	2.52 (1.60, 3.39)	2.03 (1.38, 2.82)	1.50 (1.15, 2.01)	0.046	<0.001
LDL-C (mmol/L)	4.12 (3.04, 5.42)	4.14 (3.00, 5.40)	2.71 (2.22, 3.30)	0.931	<0.001
serum IgG (g/L)	5.84 (4.39, 7.98)	7.05 (5.56, 8.98)	9.94 (8.29, 11.40)	0.016	<0.001
uric acid (μmol/L)	368.58 (101.03)	351.35 (98.03)	363.68 (90.42)	0.205	0.338
serum creatinine (μmol/L)	75.50 (62.50, 90.36)	63.89 (54.30, 78.00)	84.40 (65.50, 117.10)	<0.001	<0.001
eGFR (mL/min/1.73m^2^)	96.00 (77.50, 106.00)	105.00 (94.00, 119.00)	87.00 (63.50, 112.00)	<0.001	<0.001
hypertension n(%)	38 (38.00%)	53 (39.85%)	22 (22.00%)	0.775	0.004
systolic blood pressure (mmHg)	130.00 (115.00, 140.00)	128.00 (115.00, 140.00)	120.50 (112.50, 130.00)	0.941	0.057
diastolic blood pressure (mmHg)	80.00 (75.00, 89.00)	80.00 (70.00, 90.00)	80.00 (70.00, 85.00)	0.746	0.450
gross hematuria n(%)	0 (0.00%)	3 (2.19%)	27 (27.00%)	0.265	<0.001
microscopic hematuria (red blood cells/ul)	37.70 (22.80, 75.20)	25.00 (9.44, 66.6)	95.00 (25.00, 253.80)	0.002	<0.001
plasma IgA (g/L)	2.13 (1.58, 2.69)	2.37 (1.94, 3.10)	3.00 (2.35, 4.19)	0.030	<0.001
plasma IgA1 (g/L)	2.01 (1.41, 2.83)	2.65 (1.70, 3.16)	2.72 (2.07, 3.53)	0.079	0.180

eGFR, estimated glomerular filtration rate; LDL-C, low-density lipoprotein cholesterol.

### 4.2 Clinical Profiles of cIgAN/MN Compared to IgAN and MN

Compared to IgAN, patients with cIgAN/MN had higher 24-hour proteinuria excretion (3.96 versus 1.22 g/24h, *P*<0.001) but a similar urinary albumin-to-creatinine ratio (617.64 versus 458.04 mg/g, *P*=0.247). They showed less microscopic hematuria (25 versus 95 red blood cells/μl, *P*<0.001) and a lower incidence of gross hematuria (2.19% versus 27%, *P*<0.001). They showed better kidney function, with lower serum creatinine (63.89 versus 84.40 μmol/L, *P*<0.001) and higher eGFR level (105 versus 87 mL/min/1.73m^2^, *P*<0.001) at the time of kidney biopsy. They had a higher frequency of hypertension (39.85% versus 22%, *P*=0.004).

Compared to MN, they showed comparatively lower 24-hour proteinuria excretion but with marginal significance (3.96 versus 4.63 g/d, *P*=0.060) and presented significantly less frequency of nephrotic syndrome (47.45% versus 72%, *P*<0.001). They had a lower urinary albumin-to-creatinine ratio (617.64 versus 1552.31 mg/g, *P*=0.032). Reversely, they had higher levels of serum albumin (28.10 versus 25.35 g/L, *P*=0.025). Serum total cholesterol concentration (7.20 versus 6.85 mmol/L, *P*=0.977), low-density lipoprotein cholesterol concentration (4.14 versus 4.12 mmol/L, *P*=0.931), and the proportion of hyperlipidemia (78.63% and 82.65%, *P*=0.459) did not show a significant difference between them. Patients with cIgAN/MN also showed better kidney function, with lower serum creatinine (63.89 versus 75.50 μmol/L, *P*<0.001) and higher eGFR level (105 versus 96 mL/min/1.73m^2^, *P*<0.001) at the time of kidney biopsy compared to MN.

### 4.3 Pathological Features

Immunofluorescence showed that compared to IgAN, patients with cIgAN/MN had much weaker intensity of IgA deposition in mesangial areas (grade of intensity: 1+, 2+, 3+, 4+: 3.65% versus 0%, 60.58% versus 17%, 34.31% versus 69%, and 1.46% versus 14%, *P*<0.001). Most of the cIgAN/MN patients showed 2+ of IgA intensity, whereas most of the IgAN patients showed 3+. Similarly, compare to MN, patients with cIgAN/MN displayed weaker IgG and C3 deposition along the GBM (grade of intensity of IgG: grade 0, 1+, 2+, 3+, 4+: 2.92% versus 1%, 5.84% versus 0%, 33.58% versus 17%, 48.91% versus 69%, and 8.76% versus 13%, *P*<0.001; grade of intensity of C3: grade 1+, 2+, 3+: 38.69% versus 21%, 35.77% versus 51%, and 6.57% versus 18%, *P*<0.001). The positive rates of tissue staining for PLA2R along capillary loops were of no difference between cIgAN/MN and MN (91.30% versus 92.30%, *P*=0.639).

The light microscope showed that compared to IgAN, patients with cIgAN/MN had fewer crescents with Oxford classification (C0: 91.24% versus 44%; C1: 8.76% versus 46%; C2: 0% versus 10%, *P*<0.001). Electron microscope showed that compared to MN, there was no significant difference in the four stages described by Ehrenreich and Churg’s pathological stages in patients with cIgAN/MN (I: 50% versus 45.45%; II: 46.03% versus 49.49%; III: 3.97% versus 5.05%, *P*=0.769). Details about the pathological features are shown in **Table 2**.

**Table 2:** Pathological features of patients with MN, cIgAN/MN, and IgAN.

Pathological features	MN (n = 100)	cIgAN/MN (n = 137)	IgAN (n = 100)	*P* (cIgAN/MN vs. MN)	*P* (cIgAN/MN vs. IgAN)
immunofluorescence					
intensity of IgA in mesangial area					
0	100 (100.00%)	0 (0.00%)	0 (0.00%)	<0.001	<0.001
1	0 (0.00%)	5 (3.65%)	0 (0.00%)		
2	0 (0.00%)	83 (60.58%)	17 (17.00%)		
3	0 (0.00%)	47 (34.31%)	69 (69.00%)		
4	0 (0.00%)	2 (1.46%)	14 (14.00%)		
IgG in capillary loops					
0	1 (1.00%)	4 (2.92%)	96 (96.00%)	<0.001	<0.001
1	0 (0.00%)	8 (5.84%)	3 (3.00%)		
2	17 (17.00%)	46 (33.58%)	1 (1.00%)		
3	69 (69.00%)	67 (48.91%)	0 (0.00%)		
4	13 (13.00%)	12 (8.76%)	0 (0.00%)		
C3 in capillary loops					
0	10 (10.00%)	17 (12.41%)	96 (96.00%)	<0.001	<0.001
0.5	0 (0.00%)	9 (6.57%)	0 (0.00%)		
1	21 (21.00%)	53 (38.69%)	3 (3.00%)		
2	51 (51.00%)	49 (35.77%)	1 (1.00%)		
3	18 (18.00%)	9 (6.57%)	0 (0.00%)		
PLA2R in capillary loops (%)	92.30%	91.30%		0.639	
light microscope					
mesangial cell proliferation and matrix accumulation					
0	58 (58.00%)	4 (2.92%)	0 (0.00%)	<0.001	<0.001
1	42 (42.00%)	117 (85.40%)	30 (30.00%)		
2	0 (0.00%)	15 (10.95%)	62 (62.00%)		
3	0 (0.00%)	1 (0.73%)	8 (8.00%)		
Oxford classification of IgAN					
C0 n (%)		125 (91.24%)	44 (44.00%)		<0.001
C1 n (%)		12 (8.76%)	46 (46.00%)		
C2 n (%)		0 (0.00%)	10 (10.00%)		
electron microscope					
morphologic classification of MN					
I	45 (45.45%)	63 (50.00%)		0.769	
II	49 (49.49%)	58 (46.03%)			
III	5 (5.05%)	5 (3.97%)			
IV	0%	0%			
subepithelial electron-dense	100%	100%	0%		<0.001
mesangial electron-dense	0%	100%	100%	<0.001	

C, crescents.

### 4.4 Disease-Specific Biomarkers

#### 4.4.1 Gd-IgA1

Compared to IgAN, the level of plasma IgA in patients with cIgAN/MN was lower (2.37 versus 3.00 g/L, *P*<0.001). Although the level of plasma IgA1 was similar, they had a lower median level of Gd-IgA1 (4.00 versus 5.45 μg/ml, *P*=0.002). In contrast, patients with cIgAN/MN had a comparable level of Gd-IgA1 compared to that of MN (4.00 versus 3.64 μg/ml, *P*=0.100).

#### 4.4.2 Anti-PLA2R

**Figure 3:**
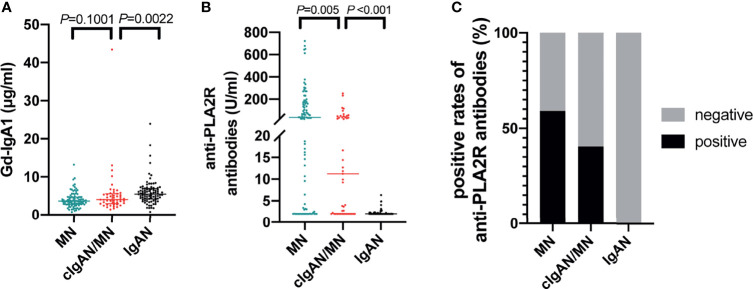
Disease-specific biomarkers detection. The comparison of the level of Gd-IgA1 **(A)**, anti-PLA2R antibodies **(B)**, and the positive rates of anti-PLA2R antibodies **(C)** among patients with IgAN, cIgAN/MN and MN.

Compared to MN, patients with cIgAN/MN had a lower frequency of plasma anti-PLA2R antibody positivity (40.43% versus 59.14%, *P*=0.036; 20U/ml as the cut-off value) and lower titers of antibody (11.23 versus 36.59 U/ml, *P*=0.005). Of certain, patients with IgAN did not have detectable anti-PLA2R antibodies in sera (**Table 3** and **Figure 3**).

**Table 3:** Disease-specific biomarkers for patients with cIgAN/MN, MN, and IgAN.

Characteristics	MN (n = 93)	cIgAN/MN (n = 47)	IgAN (n = 88)	*P* (cIgAN/MN vs. MN)	*P* (cIgAN/MN vs. IgAN)
plasma anti-PLA2R (U/ml)	36.59 (2.97, 161.04)	11.23 (2.00, 47.00)	2.00 (2.00, 2.12)	0.005	<0.001
positive rates of anti-PLA2R n (%)	55 (59.14%)	19 (40.43%)	0 (0%)	0.036	<0.001
plasma Gd-IgA1 (μg/ml)	3.64 (2.81, 4.69)	4.00 (2.94, 5.58)	5.45 (4.20, 6.88)	0.100	0.002

Gd-IgA1, galactose-deficient IgA1; anti-PLA2R, anti-phospholipase A2 receptor.

### 4.5 Disease-Specific Genetic Risk Scores (GRSs)

**Figure 4:**
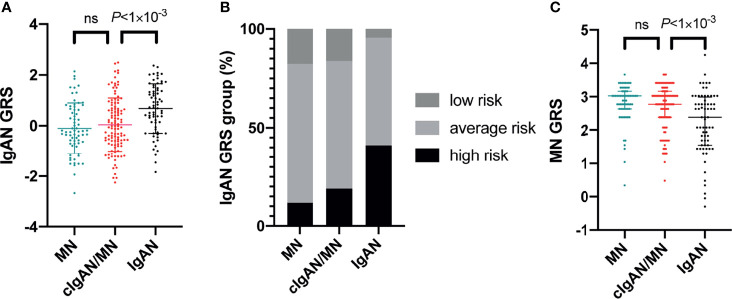
Genetic risk scores of developing IgAN or MN. The genetic risk scores of developing IgAN **(A)**, genetic risk stratification of IgAN **(B)**, and genetic risk scores of developing MN **(C)**. ns, not significant.

To determine an individual’s risk of developing IgAN and MN based on specific genetic markers, we calculated polygenic risk scores, which bridge the gap between initial discovery efforts and clinical applications for the estimation of disease risk using genetics. With the most updated genetic risk models suggested by Kiryluk et al., we genotyped 15 IgAN genetic variants and 5 MN genetic variants in 132 patients with cIgAN/MN (96.35%), 98 patients with IgAN (98%), and 96 patients with MN (96%), whose DNA were available. After quality controls, we had 111 patients with cIgAN/MN, 68 patients with IgAN, and 68 patients with MN with complete genotyping data for all the 20 SNPs (**Table 4** and **Figure 4**).

**Table 4:** GRSs of MN and IgAN in three disease groups.

Genetic risk	MN (n = 68)	MN/IgAN (n = 111)	IgAN (n = 68)	*P* (cIgAN/MN vs. MN)	*P* (cIgAN/MN vs. IgAN)
Standardized GRS of MN, median (IQR)	3.02 (2.63, 3.16)	2.77 (2.38, 3.16)	2.38 (1.54, 2.95)	0.326	<0.001
Standardized GRS of IgAN, mean (SD)	-0.10 (1.00)	0.05 (1.05)	0.68 (0.98)	0.376	<0.001
Genetic risk stratification of IgAN					
high risk	8 (11.76%)	21 (18.92%)	28 (41.18%)	0.452	0.001
average risk	48 (70.59%)	72 (64.86%)	37 (54.41%)		
low risk	12 (17.65%)	18 (16.22%)	3 (4.41%)		

GRS, genetic risk score.

#### 4.5.1 IgAN-GRSs

Compared to IgAN, patients with cIgAN/MN had lower standardized IgAN-GRSs (0.05 versus 0.68, *P*<0.001). By stratifying the IgAN-GRSs into three risk groups as reported (high >1; average -1~1; low<-1), patients with cIgAN/MN showed a significantly lower frequency of high-risk group (18.92% versus 41.18%, *P*=0.001). In contrast, this score was not significantly different from MN (0.05 versus -0.10, *P*=0.376).

#### 4.5.2 MN-GRSs

In contrast, using MN-specific-GRS, patients with cIgAN/MN did not show a significant difference (2.77 versus 3.02, *P*=0.326) compared to MN. Patients with IgAN had the lowest MN-GRS, no matter when compared to MN (2.38 versus 3.02, *P*<0.001) or cIgAN/MN (2.38 versus 2.77, *P*<0.001).

### 4.6 Follow-Up Evaluation

**Table 5** showed the medications and follow-up details for each group. At the time of renal biopsy check, the percentage of angiotensin-converting-enzyme inhibitors (ACEI) or angiotensin II receptor blockers (ARB) usage in patients with cIgAN/MN was 73.47%, which was lower than IgAN (88%, *P*=0.026), but showed no significant difference compared to MN (69.70%, *P*=0.634). 26.53% of cIgAN/MN patients had ever used glucocorticoids at the time of renal biopsy check. This percentage showed no significant difference compared with IgAN (20%, *P*=0.367) but was much lower than MN (54.55%, *P*=0.001).

**Table 5:** Follow up data from patients with MN, cIgAN/MN, and IgAN.

	MN	cIgAN/MN	IgAN	*P* (cIgAN/MN vs. MN)	*P* (cIgAN/MN vs. IgAN)
therapies	N = 99	N = 49	N = 100		
ACEI or ARBs n (%)	69 (69.70%)	36 (73.47%)	88 (88.00%)	0.634	0.026
glucocorticoids n (%)	54 (54.55%)	13 (26.53%)	20 (20.00%)	0.001	0.367
any other immunosuppressive agents n (%)	56 (56.57%)	13 (26.53%)	8 (8.00%)	<0.001	0.002
follow-up details^a^	N = 38	N = 30	N = 46		
ACEI or ARBs n (%)	28 (75.68%)	23 (85.19%)	41 (89.13%)	0.35	0.62
glucocorticoids n (%)	18 (48.65%)	5 (18.52%)	8 (17.39%)	0.013	0.9
any other immunosuppressive agents n (%)	18 (48.65%)	6 (22.22%)	3 (6.52%)	0.031	0.068
follow-up period (months)	60.00 (29.00, 108.00)	41.50 (26.00, 71.00)	54.50 (34.00, 93.00)	0.16	0.13
time-average proteinuria (g/d)	1.26 (0.62, 4.18)	0.79 (0.28, 2.24)	0.79 (0.42, 1.45)	0.08	0.93
>1.0 (g/d)	25 (65.79%)	14 (46.67%)	20 (43.48%)	0.11	0.78
≤1.00 (g/d)	13 (34.21%)	16 (53.33%)	26 (56.52%)	0.11	0.78
Complete/partial remission^b/c^ n (%)	26 (68.42%)	22 (73.33%)	44 (95.65%)	0.66	0.005
not remission n^c^ (%)	12 (31.58%)	8 (26.67%)	2 (4.35%)	0.66	0.005

a 137 cases with cIgAN/MN were systematically found from kidney pathology database without selection. However, only 1/3 of cases were regularly followed-up with a median of 4.5 years, which may limit the generality and need external validations from other centers or ethnic populations.

b Complete remission is defined as urinary protein excretion <0.5 g/24 h confirmed by two values at least one month apart, accompanied by a normal serum albumin concentration and normal serum creatinine level. Partial remission was defined as urinary protein excretion <3.5 g/24 h and reduced by at least 50% from peak values, accompanied by an improvement or normalization of the serum albumin concentration as well as stable serum creatinine.

c Categorical variables were compared using the chi-square test or Fisher’s exact test.

ACEI, angiotensin-converting enzyme inhibitors; ARB, angiotensin II receptor blockers.

Follow-up information was available from 30 cases with cIgAN/MN, 46 IgAN, and 38 MN. The percentage of ACEI or ARB usage in patients with cIgAN/MN was 85.19%, which was not significantly different compared to MN (75.68%, *P*=0.35) nor IgAN (89.13%, *P*=0.62). 18.52% of cIgAN/MN patients had ever used glucocorticoids, which also showed no significant difference compared with IgAN (17.39%, *P*=0.90) but was much lower than MN (48.65%, *P*=0.013). The level of TA-proteinuria in patients with cIgAN/MN was 0.79 g/d, which showed no significant difference between MN (1.26, *P*=0.08) and IgAN (0.79, *P*=0.93).

Comparisons across patients with or without complete follow-up data were performed, confirming no selection bias (**Supplementary Table 1**). We updated all the details among the patients who were regularly followed up until February 09^th^, 2022. The median follow-up period was 53 months. Twenty-two (73.33%) patients with cIgAN/MN achieved complete or partial remission, which was similar to MN (68.42%, *P*=0.66). 26.67% and 31.58% of patients with cIgAN/MN and MN showed no proteinuria remission. However, the percentages of glucocorticoids and other immunosuppressive agents in patients with cIgAN/MN were lower than those in MN. We assume milder pathology lesions and low anti-PLA2R titers in patients with cIgAN/MN might explain this difference.

**Figure 5:**
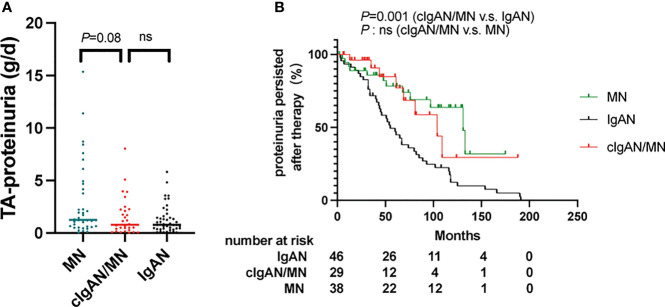
**(A)** The comparison of the time-average proteinuria among patients with IgAN, cIgAN/MN and MN. **(B)** Proteinuria persisted means patients could not achieve complete or partial proteinuria remission during follow-up, which was were calculated according to the Kaplan-Meier method (log-rank test). ns, not significant.

Kaplan-Meier analysis (**Figure 5**) showed that the cumulative incidence of complete or partial remission was similar between patients with cIgAN/MN and MN or IgAN. Compared to IgAN, the cumulative incidence of persistent proteinuria after therapy in patients with cIgAN/MN was higher than in IgAN (*P*=0.001).

## 5 Discussion

The present study mainly shows the clinical and pathological data about 137 patients with cIgAN/MN. Additionally, disease-specific biomarkers and genomics analysis were checked for a better understanding of the pathogenesis of cIgAN/MN.

From 1983, an increasing number of studies reported the concurrent of IgAN and MN in the same patient. Doi et al. firstly described their clinical and pathological characteristics, showing that cIgAN/MN had features from both IgAN and MN (5). Subsequently, more cases had been noticed in the clinic, and the reported patients had hematuria and nephrotic range proteinuria (6, 8–10). Magil et al. also showed an isolated case, suggesting that cIgAN/MN might be associated with hepatitis B surface antigenemia (7). Other related types of research showed that IgAN and MN occurred separately in one patient after some years’ interval (20, 21). Our previous case series summarized the clinical and pathological profiles of 26 patients with cIgAN/MN. The results showed that patients with cIgAN/MN displayed similar clinical features with MN patients and milder pathological lesions than IgAN patients (12). Besides, the prognosis of patients with cIgAN/MN was better than that in MN (11). However, due to a relatively small sample size and incomplete analysis, the pathogenesis of cIgAN/MN remained unclarified. In this study, we showed clinical-pathological features in patients of cIgAN/MN with a larger sample size.

The present cohort of patients with cIgAN/MN, to our knowledge, is the largest published to date. Our results showed that cIgAN/MN has common features of the two glomerular diseases. Firstly, the level of proteinuria and eGFR, the percentage of nephrotic syndrome, hypertension are much higher than IgAN. Whereas the rate of gross hematuria, the level of urinary microscopic hematuria, and plasma IgA in patients with cIgAN/MN are much lower than IgAN. Patients with cIgAN/MN showed fewer crescents than IgAN. Thus the clinical and pathological features of patients with cIgAN/MN are not in accordance with those of IgAN patients. Secondly, patients with cIgAN/MN and MN have comparable proteinuria levels, hyperlipidemia rates, serum total cholesterol levels, serum low-density lipoprotein cholesterol levels, hypertension rates, and gross hematuria rates. Pathological features also indicated no significant differences in the positive rates of PLA2R along capillary loops and pathological stages of MN between patients with cIgAN/MN and MN. These clinical and pathological features between patients with cIgAN/MN and MN patients were much similar. Thus, we suggest priority be given to MN when facing cIgAN/MN.

**Figure 6:**
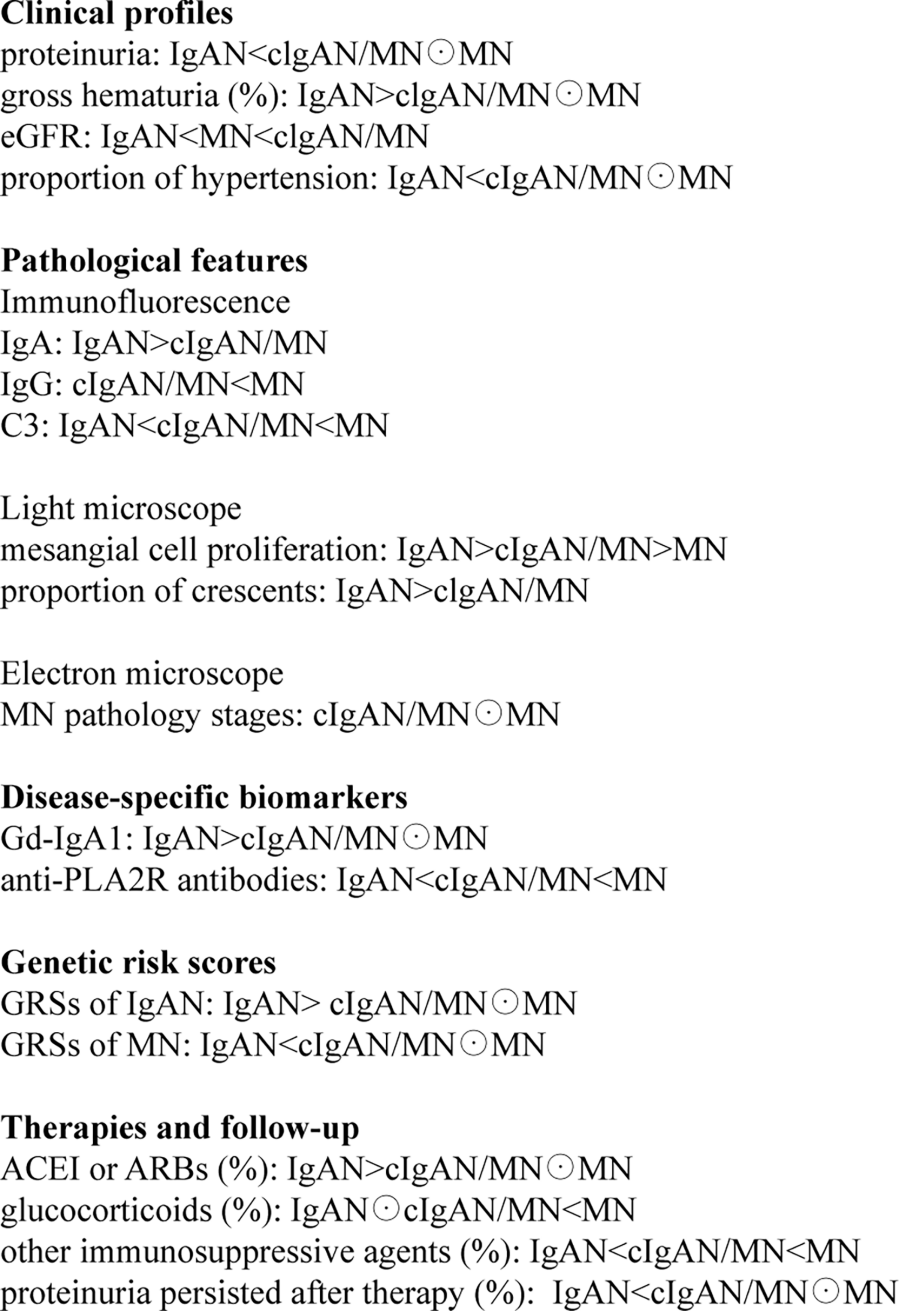
The symbol “<“ or “>“ indicates that the specific item is lower or higher than the other group, respectively. Moreover, the symbol “⊙” means no statistical difference between the two groups.

Moreover, we used disease-specific biomarkers and a genomics approach to shed some light on its pathogenesis. The plasma level of Gd-IgA1 in patients with cIgAN/MN is much lower than IgAN but had no significant difference with MN. Thus, Gd-IgA1 seems not to be a significant pathogenic factor in cIgAN/MN. On the other hand, nearly 40.43% of patients with cIgAN/MN had detectable plasma levels of anti-PLA2R antibodies. Although the plasma level of anti-PLA2R antibodies in cIgAN/MN is much lower than that in MN, anti-PLA2R antibodies may at least in part contribute to the pathogenesis of cIgAN/MN. To gain more insights into the pathogenesis of cIgAN/MN from a genetic perspective, we genotyped the majority of the subjects examined. The genetic risk score of developing IgAN in patients with cIgAN/MN is much lower than IgAN. Stratified analyses also showed that the high-risk group of developing IgAN in patients with cIgAN/MN is much lower than IgAN. Intriguingly, after calculating the genetic risk scores of developing MN, we did not observe a significant difference between patients with cIgAN/MN and MN. Thus, the genetic background of cIgAN/MN resembles that of MN. Therefore, the present study may demonstrate that cIgAN/MN seems to be just concurrent, not the overlap syndrome of IgAN and MN. cIgAN/MN may result from superimposed MN on a background of mild IgAN or IgA deposition (summarized in **Figure 6**).

Despite a relatively large-scale study on cIgAN/MN, we should note some limitations. First, the pathogenesis of cIgAN/MN is still not clear, but disease-specific markers in the circulatory system may be highlighted in disease differentiation. Second, patients with cIgAN/MN enrolled in the present study are diagnosed simultaneously by renal biopsy. We cannot rule out the possibility that IgAN or MN may superimpose on the basis of preexisting one of the other glomerular diseases. Third, our follow-up data are not complete. Only one-third of the patients enrolled from pathology dataset had been regularly followed-up. The composite endpoint, such as the decline rate in eGFR and the receipt of renal replacement therapy, should be further recorded in long-term follow-up. Fourth, as a growing number of cases will be discovered in the clinic, additional research with larger sample sizes is still warranted. More studies focusing on other biomarkers related to underlying mechanisms, such as T cells and activation of the complement system, will also be conducted (22–26). Fifth, we mainly recruited participants of Chinese Han ethnicity. IgA deposition was different among various ethnicities (27–30). Replication from different ethnicities or different populations was needed for similar studies.

In conclusion, although MN is concurrent with IgA deposition and mesangial proliferation in pathology, from the clinical, diagnostic, and prognostic point of view, cIgAN/MN is more likely to be MN accompanied by IgA deposition and may not be an independent disease.

## Data Availability Statement

The original contributions presented in the study are included in the article/**Supplementary Material**. Further inquiries can be directed to the corresponding authors.

## Ethics Statement

All the study protocols complied with the principles of the Declaration of Helsinki and were approved by the Ethics Committee of Peking University First Hospital (Institutional Review Board number: 2021[Y148]). The patients/participants provided their written informed consent to participate in this study.

## Author Contributions

HZ and X-JZ conceived and designed the study. X-JZ, S-FS, L-JL, J-CL, J-WH, D-FC, and LZ collaborated in patient recruitment, data acquisition, and organization. J-WH, PC, YL, XZ, and PH performed the laboratory analyses. J-WH, D-FC, Y-NW, and TG analyzed the data. X-JZ and J-WH made the figures. J-WH, D-FC, X-JZ, and HZ drafted and revised the manuscript. All authors contributed to the article and approved the submitted version.

## Funding

Support was provided by Beijing Natural Science Foundation (Z190023); National Science Foundation of China (82022010, 82131430172, 81970613, 82070733, 82000680, 82070731); Academy of Medical Sciences --Newton Advanced Fellowship (NAFR13\1033); Fok Ying Tung Education Foundation (171030); Beijing Nova Program Interdisciplinary Cooperation Project (Z191100001119004); CAMS Innovation Fund for Medical Sciences (2019-I2M-5-046). The funders had no role in study design, data collection and analysis, decision to publish, or preparation of the manuscript.

## Conflict of Interest

The authors declare that the research was conducted in the absence of any commercial or financial relationships that could be construed as a potential conflict of interest.

## Publisher’s Note

All claims expressed in this article are solely those of the authors and do not necessarily represent those of their affiliated organizations, or those of the publisher, the editors and the reviewers. Any product that may be evaluated in this article, or claim that may be made by its manufacturer, is not guaranteed or endorsed by the publisher.
