# Corrigendum: Concurrent IgA nephropathy and membranous nephropathy, is it an overlap syndrome?

**DOI:** 10.3389/fimmu.2023.1218427

**Published:** 2023-06-09

**Authors:** Jia-Wei He, Dong-Feng Cui, Xu-Jie Zhou, Pei Chen, Yang Li, Xue Zhang, Yan-Na Wang, Ting Gan, Li-Jun Liu, Su-Fang Shi, Li Zhu, Ping Hou, Ji-Cheng Lv, Hong Zhang

**Affiliations:** ^1^ Renal Division, Peking University First Hospital, Peking University Institute of Nephrology, Key Laboratory of Renal Disease, Ministry of Health of China, Key Laboratory of Chronic Kidney Disease Prevention and Treatment (Peking University), Ministry of Education, Beijing, China; ^2^ Renal Division, The Third People’s Hospital of Zhengzhou, Zhengzhou, China

**Keywords:** IgA nephropathy, primary membranous nephropathy, galactose-deficient IgA1, anti-phospholipase A2 receptor, polygenic risk score

In the published article, there was an error in the legend for **Figure 5** as published. Part (A) incorrectly read “The comparison of the total-average proteinuria” instead of “The comparison of the time-average proteinuria”. The corrected legend appears below.

**Figure 5:**
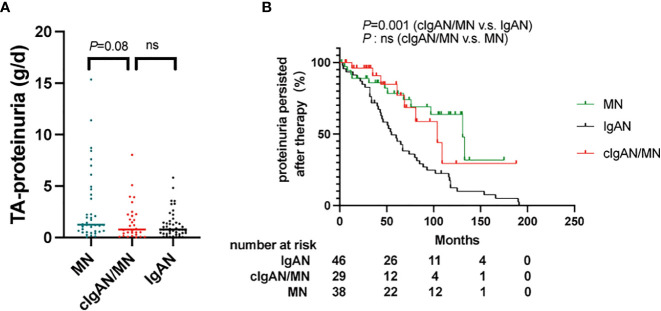
**(A)** The comparison of the time-average proteinuria among patients with IgAN, cIgAN/MN and MN. **(B)** Proteinuria persisted means patients could not achieve complete or partial proteinuria remission during follow-up, which was were calculated according to the Kaplan-Meier method (log-rank test). ns, not significant.

In the published article, there was also a word expression error.

A correction has been made to **Results**, *Follow-Up Evaluation*, paragraph number 4. This sentence previously stated:

“Compared to IgAN, the cumulative incidence of persistent proteinuria after therapy in patients with cIgAN/MN was lower than in IgAN (*P*=0.001).”

The corrected sentence appears below:

“Compared to IgAN, the cumulative incidence of persistent proteinuria after therapy in patients with cIgAN/MN was higher than in IgAN (*P*=0.001).”

The authors apologize for these errors and state that they do not change the scientific conclusions of the article in any way. The original article has been updated.

