# Supplementary material for: Concurrent IgA Nephropathy and Membranous Nephropathy, Is It an Overlap Syndrome?
Source: Front Immunol. 2022 Mar 11;13:846323. doi: 10.3389/fimmu.2022.846323 (PMC8961684; doi:10.3389/fimmu.2022.846323)
Supplement: Supplementary file 1 [file Table_1.docx]

Supplementary Material

# Supplementary Table 1. Comparisons of the patients with or without follow-up data

|  | MN | | | cIgAN/MN | | | IgAN | | |
| --- | --- | --- | --- | --- | --- | --- | --- | --- | --- |
| Characteristics | not follow-up (n=62) | follow-up (n=38) | *P* values | not follow-up (n=107) | follow-up (n=30) | *P* values | not follow-up (n=54) | follow-up (n=46) | *P* values |
| age (y) | 53.50 (43.00, 64.00) | 47.50 (38.00, 61.00) | 0.082 | 43.00 (35.00, 54.00) | 48.50 (38.00, 59.00) | 0.105 | 34.50 (28.00, 42.00) | 35.50 (31.00, 44.00) | 0.304 |
| female n(%) | 26 (41.94%) | 16 (42.11%) | 0.987 | 52 (48.60%) | 11 (36.67%) | 0.247 | 26 (48.15%) | 21 (45.65%) | 0.803 |
| nephrotic syndrome n(%) | 47 (75.81%) | 25 (65.79%) | 0.279 | 53 (49.53%) | 12 (40.00%) | 0.355 | 8 (14.81%) | 2 (4.35%) | 0.082 |
| proteinuria (g/24h) | 4.78 (3.98, 8.37) | 4.56 (2.25, 7.01) | 0.460 | 4.55 (2.35, 6.17) | 2.90 (1.75, 5.06) | 0.048 | 1.34 (0.67, 3.00) | 0.83 (0.54, 1.78) | 0.063 |
| albumin-to-creatinine ratio (mg/g) | 1.56 (0.48, 3.06) | 1.22 (0.32, 3.07) | 0.692 | 0.34 (0.21, 0.93) | 0.72 (0.34, 2.14) | 0.151 | 0.52 (0.24, 1.13) | 0.42 (0.18, 0.71) | 0.342 |
| serum albumin (g/L) | 24.70 (21.60, 29.50) | 27.20 (22.60, 31.10) | 0.138 | 27.00 (21.50, 33.00) | 29.45 (25.10, 35.10) | 0.055 | 37.60 (32.90, 41.80) | 38.65 (36.50, 41.70) | 0.182 |
| hyperlipidemia n(%) | 52 (86.67%) | 29 (76.32%) | 0.187 | 73 (81.11%) | 19 (70.37%) | 0.232 | 19 (35.19%) | 11 (24.44%) | 0.247 |
| total cholesterol (mmol/L) | 7.08 (6.20, 8.80) | 6.62 (5.33, 8.14) | 0.091 | 7.43 (6.01, 8.75) | 6.82 (4.81, 7.58) | 0.134 | 4.80 (4.12, 5.81) | 4.53 (4.03, 5.04) | 0.136 |
| triglycerides (mmol/L) | 2.50 (1.48, 3.42) | 2.52 (1.62, 3.34) | 0.538 | 1.98 (1.31, 2.94) | 2.30 (1.47, 2.82) | 0.430 | 1.50 (1.18, 2.15) | 1.43 (0.95, 2.00) | 0.407 |
| LDL-C (mmol/L) | 4.27 (3.38, 5.78) | 3.46 (2.79, 5.21) | 0.041 | 4.50 (3.21, 5.67) | 3.36 (2.31, 4.65) | 0.019 | 2.76 (2.32, 3.47) | 2.69 (2.19, 3.18) | 0.458 |
| serum IgG (g/L) | 5.90 (4.04, 7.86) | 5.73 (4.73, 8.10) | 0.371 | 6.88 (5.54, 8.14) | 8.12 (5.56, 9.37) | 0.050 | 9.09 (7.80, 10.80) | 10.50 (9.77, 12.10) | 0.005 |
| uric acid (μmol/L) | 362.18 (101.79) | 378.68 (100.32) | 0.434 | 341.05 (98.75) | 381.90 (90.69) | 0.048 | 339.26 (85.33) | 392.35 (88.63) | 0.003 |
| serum creatinine (μmol/L) | 72.45 (63.00, 86.00) | 78.85 (60.30, 93.00) | 0.534 | 62.00 (53.30, 76.00) | 69.19 (56.60, 85.19) | 0.210 | 77.30 (61.00, 91.90) | 95.48 (76.60, 128.40) | 0.003 |
| eGFR (mL/min/1.73m^2^) | 95.00 (83.00, 105.00) | 97.50 (74.00, 108.00) | 0.642 | 108.00 (96.00, 120.00) | 103.00 (89.00, 107.00) | 0.071 | 99.00 (78.00, 115.00) | 75.00 (61.00, 92.00) | 0.002 |
| hypertension n(%) | 25 (40.32%) | 13 (34.21%) | 0.541 | 41 (39.42%) | 12 (41.38%) | 0.849 | 14 (25.93%) | 8 (17.39%) | 0.304 |
| systolic blood pressure (mmHg) | 130.00 (110.00, 140.00) | 126.00 (120.00, 140.00) | 0.730 | 130.00 (118.00, 140.00) | 123.00 (110.00, 132.00) | 0.101 | 120.00 (115.00, 130.00) | 123.50 (111.00, 130.00) | 0.909 |
| diastolic blood pressure (mmHg) | 80.00 (75.00, 90.00) | 80.00 (75.00, 85.00) | 0.297 | 80.00 (70.00, 90.00) | 75.00 (70.00, 80.00) | 0.018 | 80.00 (70.00, 85.00) | 78.00 (70.00, 82.00) | 0.235 |
| gross hematuria n(%) | 0 (0%) | 0 (0%) | / | 2 (1.87%) | 1 (3.33%) | 0.527 | 17 (31.48%) | 10 (21.74%) | 0.274 |
| microscopic hematuria (RBCs/ul) | 38.25 (23.00, 68.90) | 37.70 (18.10, 86.90) | 0.683 | 20.00 (4.50, 69.95) | 25.00 (13.50, 53.40) | 0.325 | 81.70 (24.60, 272.00) | 101.00 (39.30, 228.45) | 0.483 |
| plasma IgA (g/L) | 2.13 (1.58, 2.79) | 2.10 (1.58, 2.48) | 0.937 | 2.34 (1.94, 2.98) | 2.70 (1.68, 3.16) | 0.607 | 2.89 (2.19, 3.62) | 3.41 (2.49, 4.39) | 0.090 |
| plasma IgA1 (g/L) | 2.01 (1.38, 2.66) | 2.00 (1.63, 3.27) | 0.725 | 2.69 (1.81, 2.87) | 2.56 (1.70, 3.16) | 0.966 | 2.61 (2.07, 3.33) | 2.83 (2.08, 3.81) | 0.265 |
